# Short-Term Complications of Hemorrhoidectomy in Outpatient and Inpatient Operations in Shiraz, Southern Iran

**Published:** 2011-04-01

**Authors:** A Keshtkaran, S V Hosseini, L Mohammadinia

**Affiliations:** 1 School of Management and Information Science, Shiraz University of Medical Sciences, Shiraz, Iran; 2 Laparascopy Research Center,Shiraz University of Medical Sciences, Shiraz, Iran; 3 Stem Cell and Transgenic Technology Research Center, Shiraz University of Medical Sciences, Shiraz, Iran; 4 Health Service Management, Faghihi Hospital, Shiraz University of Medical Sciences, Shiraz, Iran

**Keywords:** Complications, Hemorrhoidectomy, Hospitalization, Outpatient, Inpatient

## Abstract

**Background:**

Today, hospitals and patients are both willing to benefit from outpatient services. Considering limits of supply, it seems that there is a need to run productive management in offering health services to prevent wasting of supplies and facilities. This study compares the complications caused by hemorrhoidectomy in outpatient and inpatient operations.

**Methods:**

In a cross-sectional study during 1.5 years, 208 patients without any background disease were enrolled. They were randomly allocated into two groups (inpatient and outpatient) and interviewed within two weeks after surgical operations. The data were collected through a questionnaire and physical examination. The complications in the two groups of operating theater of hospital and clinic were then compared regarding sex, occupation, education and etc.

**Results:**

One week after the surgical operation, the patients in the hospital operating theater showed significantly a better healing recovery of their wound. Other complications such as pain, hemorrhage, infection, inflammation, involuntary emission of feces and gas indicated no significant difference between the two groups. After 2 weeks, more pain was noticed in patients in the operating theaters of the hospital and in clinics, there was more infection visible. The hemorrhage, inflammation, wound healing, involuntary emission of feces and gas did not indicate a significant difference between the operating theater of hospital and the clinic. There was no significant difference regarding the patients' satisfaction in the two groups.

**Conclusion:**

We recommend that for optimized use of supplies and equipments in operating theaters and to lower the cost and shorten queue of patients, grade 2 hemorrhoids are performed in the operating theater of clinics considering sterilization and safety procedures.

## Introduction

Today, performing outpatient operation has become very popular worldwide.[1][2] In United States, over 60% of non-emergency surgical operations are performed in an outpatient method.[3] Outpatient surgical operations in health economy are associated with a lower cost and have other advantages such as decrease in hospital infections.[2] Furthermore, it has advantages for patients and medical personnel tool. In United States, at least one million people annually suffer from hospital infections.[4] In some studies, half of individuals older than 50 years indicated symptoms of a hemorrhoid disease and generally hemorrhoidectomy was operation of choice in the anal area.[5] Since hemorrhoidectomy can be treated in both outpatient and inpatient departments,[6] this study aims to compare the outcome in clinic and hospital settings.

## Materials and Methods

In a cross-sectional study during 1.5 years, 208 patients aged between 16 to 80 years referring to Faghihi Hospital and Shahid Motahari Clinic in Shiraz, southern Iran for hemorrhoidectomy entered the study. They were randomly allocated into two groups undergoing operation in a hospital and clinic. The data the patients were recorded during surgical operation and one or two weeks after attending the physician’s office. They were all examined and interviewed. Hemorrhoidectomy was performed in two types of cases with sphincteratomy and without cutting sphincter in grade 2, 3, and 4 hemorrhoid patients who did not respond to outpatient treatment. Internal and external hemorrhoids were both considered. All patients in the clinic were surgically operated with local anesthesia and only 14/6% of those in the operating theater of the hospital were operated with local anesthetic and the remaining were operated with general anesthesia. The level of pain, amount of hemorrhage, infection, inflammation, involuntary emission of feces and gas, healing of wound and level of satisfaction from the operation were determined. The severity of these complications were evaluated and scored on the Likert scale of very much, much, average, little and none levels. The score of one indicated highest and five indicated the least amount of severity. The patients were evaluated by filling out a questionnaire, one week and two weeks after the surgical operation. T test was used to analyze quantitative and Chi-Square test for qualitative variables. One-way Analysis of Variance was also performed.

## Results

One hundred and three patients were operated in the operating theater of hospital and 105 patients in the clinic. The average age of the patients was 40 years old. Sixty five percent of patients in the operating theater of hospital were male and in the clinic, 51.4% were male. Hemorrhoidectomy was performed for 54.4% of patients in the operating theater of hospital and 51.4% of the clinic by cutting the sphincter. Twenty three percent of patients had undergone hemorrhoidectomy before (13% in the operating theater of hospital and 10% in the clinic).No significant difference was noticed between the level of satisfaction among the patients of the operating theater of hospital and the clinic. One week after the operation, the amount of pain, hemorrhage, infection, inflammation, involuntary emission of feces and gas in the operating theater of the hospital and clinic were not statistically different (Table 1).

**Table 1:** Comparson of the average score of severity (1-5)of complications of hemorrhoidectomy, one week after the operation in patients operated in the operating theater of Faghihi Hospial with Shiraz Motahhari Clinic, 2007-2008.

**Descriptive Statistics Complicationsa**	**Place of Treatment**	**Average**	**Standard Deviation**	**P value**
Pain	Operating Theater of hospital	1.57	1.03	0.197
	Clinic	1.77	1.17	
Hemorrhage	Operating Theater of hospital	3.58	1.08	0.382
	Clinic	3.71	1.09	
Infection	Operating Theater of hospital	4.39	0.92	0.163
	Clinic	4.18	1.20	
Inflammation	Operating Theater of hospital	3.74	1.01	0.459
	Clinic	3.63	1.11	
Involuntary emission of feces	Operating Theater of hospital	4.81	0.54	0.426
	Clinic	4.73	0.75	
Involuntary emission of wind from anus	Operating Theater of hospital	4.43	0.95	0.464
	Clinic	4.32	1.06	
Wound Recovery	Operating Theater of hospital	3.26	0.70	0.035
	Clinic	3.02	0.93	

^a^ It is noticeable that the level of significant of all tests is equal or less than 0.05

One week after the operation, wound healing in patients of the clinic was less and a decrease in pain was seen in 82% of the patients in the hospital and 75% in the clinic. One week after the operation, in 41% of the patients in the hospital and 28% of those in the clinic, the amount of hemorrhage was from average to high. Two weeks after the operation, there was no significant difference in the complications of the patients of the operating theater of the hospital and clinic. A significant difference was visible regarding pain and infection two weeks after the surgery in the hospital and clinic while pain was more in patients of the hospital but infection was more among patients of the clinics (Table 2).

**Table 2:** Comparson of the average score of severity (1-5) of complications of hemorrhoidectomy, two weeks after the operation of the patients in Faghihi Hospital and Motahhari Clinic of Shiraz in 2007-2008.

**Descriptive Statistics Complicationsa**	**Place of Treatment**	**Average**	**Standard Deviation**	**P value**
Pain	Operating Theater of hospital	3.10	1.23	0.008
	Clinic	3.57	1.24	
Hemorrhage	Operating Theater of hospital	4.58	0.63	0.92
	Clinic	4.59	0.64	
Infection	Operating Theater of hospital	4.70	0.62	0.008
	Clinic	4.40	1	
Inflammation	Operating Theater of hospital	4.42	0.90	0.497
	Clinic	4.34	0.90	
Involuntary emission of feces	Operating Theater of hospital	4.96	0.23	0.812
	Clinic	4.95	0.30	
Involuntary emission of wind from anus	Operating Theater of hospital	4.76	0.60	0.133
	Clinic	4.60	0.88	
Wound Recovery	Operating Theater of hospital	3.77	0.82	0.912
	Clinic	3.80	0.95	

^a^ It is noticeable that the level of significant of all tests is equal or less than 0.05

Twenty three percent of our patients had a previous experience of hemorrhoidectomy in two centers. The waiting period for hemorrhoidectomy in the operating theater of the hospital was more than the clinic; the ability to return to daily activities in both periods (one week and two weeks after the surgical operation) for the patients of clinic was faster than those of the operating theater of the hospital. The relapse of disease was seen in 4% of patients. Ninety percent of patients in both groups did not need to undergo hemorrhoidectomy for another time.

## Discussion

In the study by Havran et al. (2007), none of the patients needed hemorrhoidectomy for the second time and only 3.7% of the patients needed to be hospitalized.[7] In a research by Khoshkalam et al. (2004), the most important reason for re-hospitalization was the side effect after the operation for 72% of patients, infection after the operation for 19.5% and relapse of disease in 8.5%. Considering the high risk of re-hospitalization resulting from the complications and infection after the operation about 91.5%, more precise studies are required in quality management of surgical operations and sterilization of the operating theater of the hospital and related units[8].

Among patients of the operating theater of the hospital, 51.5% reported a grade 3 hemorrhoid and 63.8% of them in the clinic had a grade 2 hemorrhoid. Therefore, patients with hemorrhoid of higher grades are preferred to be treated in the operating theater of hospital due to seriousness of the disease as under general anesthesia, patients take relaxant medicines and are not conscious which is more suitable for the surgeon. Some studies indicated that an unconscious patient has no movement and reflex and the surgeon is highly satisfied with general anesthesia while the satisfaction of patients undergoing local anesthesia is also favorable as it is an easy method without any nausea and vomiting and systematic effects are scarce too.[9] Ninety one percent of patients in operating theater of the hospital were not able to do their routine works in about one week after the operation while this rate was 78% in the clinic and the patients operated in the clinics returned to their normal life faster.[2] Ghobadi et al. (2007) believes that patients undergoing general anesthesia return to normal life faster than those with local anesthesia in outpatient operations.[10]

In the second week after hemorrhoidectomy, considering the ability to do the routine works, both groups were similar.

There was a difference between the treatment results of the operating theater of Faghihi hospital and clinic regarding waiting period, ability to do daily activities after the operation, the period of recovery one week after the operation, and two weeks after the operation, and emission control of gas (p<0.05). This comparison showed that waiting period for hemorrhoidectomy in the operating theater of the hospital was more than the clinic; the ability to return to daily activities in both periods (one week and two weeks after the surgical operation) for the patients of clinic was faster than those of the operating theater of the hospital. Several studies compared the complications and pain after the operation with general anesthesia and local anesthesia, and most of these studies have shown less pain and lower systematic complications in the group of local anesthesia,[11][12] but the rest could not indicate such benefits.[13][14] In the study by Krioluck et al. (2005), there was no significant relation between inpatients and outpatients in aspects of pain after the operation, nausea, re-hospitalization, complications and patient's complaints.[15]

Most of the studies in the recent years have compared common hemorrhoidectomy and hemorrhoidectomy by means of modern instruments including Chitam et al. (2000), Laves et al. (2004), Havran et al. (2007) and Johnson et al. (2006) studies. Several authors have also studied the satisfaction and complications in these states.[7][16][17][18][19] The study by Johnson et al. (2006) states that the satisfaction and fecal control in hemorrhoidectomy in the open method is more than the closed one[18] while Hou et al. (2007) like other researchers, observed no significant difference between the patient's satisfaction and pain after the operation in the two methods.[19]

The results of this research open a new point of view to the surgeons, directors and those planning health services and considering advantages of outpatient treatment, physicians are recommended to perform hemorrhoidectomy surgeries in clinics more.
